# High throughput screening of key functional strains based on improving tobacco quality and mixed fermentation

**DOI:** 10.3389/fbioe.2023.1108766

**Published:** 2023-01-13

**Authors:** Cai Wen, Qianying Zhang, Pengcheng Zhu, Wanrong Hu, Yun Jia, Shuanghong Yang, Yang Huang, Zhen Yang, Zhishun Chai, Tianyuan Zhai, Yu Cao, Dongliang Li

**Affiliations:** China Tobacco Sichuan Industrial Co., Ltd., Chengdu, China

**Keywords:** fermentation optimization, flue-cured tobacco, co-cultivation, industrial fermentation technology, quality improvement and aroma enhancement

## Abstract

**Background:** Tobacco alcoholization is an important step in increasing the quality of tobacco leaf, which may convert a portion of low-grade tobacco leaves into useable product, however this may take to 2–3 years. The addition of exogenous microorganisms to tobacco leaves and treating them by biological fermentation can shorten the maturation time of tobacco leaves, and improve the quality and applicability of low-grade tobacco leaves

**Methods:** Several strains were screened from low-grade tobacco by flow cytometry, including the bacteria *Bacillus amyloliticus*, with starch degradation ability and *Bacillus kochii*, with protein degradation ability, and the fungus *Filobasidium magnum* with lipid oxidase ability, and were inoculated onto tobacco leaves, both individually and in combination, for solid-state fermentation

**Results:** The greatest improvement in tobacco quality was observed when strains 4# and 3# were applied at a ratio of 3:1. The Maillard reaction products, such as 2-amyl furan, 1-(2-furanmethyl) -1 h-pyrrole, furfural and 2, 5-dimethylpyrazine, were significantly increased, by up to more than 2 times. When strains F7# and 3# were mixed at a ratio of 3:1, the improvement of sensory evaluation index was better than that of pure cultures. The increase of 3-(3, 4-dihydro-2h-pyrro-5-yl) pyridine, β -damasone and benzyl alcohol was more than 1 times. The increase of 2-amyl-furan was particularly significant, up to 20 times

**Conclusion:** The functional strains screened from tobacco leaves were utilized for the biological fermentation of tobacco leaves, resulting in the reduction of irritation and an improvement in quality of final product, showing a good potential for application.

## 1 Introduction

Differences in climate, soil and planting agronomic measures, all affect tobacco leaf quality. Freshly harvested tobacco leaves have the disadvantages of being heavily green and irritating, and as such they must be alcoholized (Ma et al., 2020). The aging process of tobacco leaves is an important part of the quality improvement of raw materials in cigarette production (Liu, 2020). Aging occurs after redrying, a process that respectively passivates or inactivates the microorganisms and enzyme activities on tobacco leaves. At the same time, in order to prevent mildewing of tobacco leaves during the aging process, their water content is generally controlled at 14%–20% (Guo et al., 2020). Therefore, the total amount of microorganisms in tobacco leaves during the aging process is small and their activity is low, resulting in low microbial efficiency (Zeng et al., 2009). Under the combined action of microorganisms, enzymes and chemical action, the chemical composition of tobacco leaves changes slowly, resulting in an alcoholization process that can take 1–3 years (Wang et al., 2017). Some tobacco leaves do not reach the expected quality after alcoholization. Therefore, how to improve the efficiency of tobacco leaf alcoholization in industrial production and shorten the processing time is a key goal of the current alcoholization process reform.

Numerous studies have shown that spraying beneficial microorganisms on the surface of tobacco leaves can significantly shorten the fermentation and curing time. Xie et al. pointed out that adding *Bacillus subtilis* in the fermentation process of flue-cured tobacco can shorten the artificial fermentation time to 8 days (Xie et al., 1998). Therefore, the use of artificial fermentation for tobacco leaves can greatly improve the efficiency of alcoholization and shorten the curing time of tobacco leaves (Wang, 2006). Through the study of microbial diversity in tobacco leaves, it was found that many different types of microorganisms, and that their functions were closely related to the production of flavor precursors and flavor substances. Among them, bacteria played a major role (Yang et al., 2014). Bacteria had higher functional abundance in amino acid metabolism, carbohydrate metabolism and secondary metabolite metabolism, while fungi had higher functional abundance in fatty acid metabolism (Zhang Y. J. et al., 2022). However, due to the low moisture content of tobacco leaves, microorganisms were in a dormant state and the efficiency was low. The major quality defects of low-grade tobacco leaves are irritation, peculiar smell and lack of aroma, etc. (Liu et al., 2013). Usually, the irritation and green gas in tobacco leaves are mostly caused by the high content of high molecular substances in tobacco leaves. The lack of flavor and aroma is closely related to the low content and limited range of aroma substances. Therefore, in this study we screened for functional microbes to strengthen the degradation of high molecular weight substances and promote the formation of aroma substances.

Excessive starch and protein content in flue-cured tobacco is one of the main reasons for irritation and odor (Shen et al., 2020). The starch content in flue-cured tobacco after redrying generally ranges from 4%–6%, but could be reduced to 3% by degradation. At the same time, the degradation of starch helps to increase the content of soluble polysaccharides or reducing sugars, thereby improving the sweetness and aftertaste of cigarettes (Lin et al., 2020). The protein content of tobacco varies greatly with the maturity of fresh tobacco. After the redrying process, it usually drops to about 5%. Excessive protein content may cause stronger irritation and odor. When the cigarette burns, it will produce a pungent burnt feather odor and poor flammability, which seriously affects the quality of tobacco leaves and the health of smokers (Xie et al., 2009). Therefore, the quality of tobacco leaves can be greatly improved by adding functional microorganisms in the alcoholization process to further degrade starch and protein (Xue et al., 2022). The increase of reducing sugar and amino acid content can also promote the formation of Maillard reaction products under certain conditions, and enhance the flavor of cigarettes by increasing the alcohol and aftertaste after burning (Gao et al., 2015).

In previous studies, microorganisms were used to accelerate the degradation of proteins in tobacco leaves. These microorganisms included *Bacillus*, *Micrococcus* and other thermophilic bacteria (Zhou et al., 2002). Li et al. applied *Bacillus cereus* to cigar tobacco stacking fermentation, and could reduce the protein content in tobacco leaves by 13.53% (Li et al., 2012). Feng et al. utilized the bacterial strain F-Y-11 (*Bacillus*) as a bacterial agent to ferment tobacco, and the protein degradation rate was 29.32% (Feng et al., 2012). In the study of *Micrococcus* and thermophilic bacteria, Feng et al. also prepared a compound biological agent using *Micrococcus* and *Gluconobacter* to produce aroma components in fresh tobacco leaves under high temperature baking conditions, which effectively improved the quality of fresh tobacco leaves (Feng et al., 2021). Therefore, screening for and utilization of functional microorganisms with high-yield amylase and protease would help to promote the reduction of starch and protein content in tobacco leaves, reduce irritation, and improve quality.

In 1953, Tamayo carried out experimental inoculation of tobacco leaves to increase their aroma (Tamayo, 1978). Since then, studies have confirmed that *Bacillus* and *Micrococcus* can improve the aroma of tobacco leaves, and many other aroma-producing microorganisms have been reported (Xue et al., 2019). For example, *Pantoea* have been shown to degrade carotenoids to produce important aroma substances in tobacco, and some actinomycetes possess the ability to catalyze ferulic acid to produce vanillin (Wang et al., 2009). Yu et al. found that tobacco leaves inoculated with *Klebsiella pneumoniae*, *Bacillus simianus* V16 or *Bacillus thermophilus* rapidly produced a pleasant aroma (Yu et al., 2016). Through analysis of the chemical composition of tobacco leaves, it was found that the content of aldehydes, ketones, phenols, alcohols, acids, heterocycles and other aroma components in tobacco leaves increased by varying degrees during fermentation (Zhou et al., 2014). Like many plants, terpenoids are important flavor precursors in flue-cured tobacco. Carotenoids (including lutein, lycopene, etc.) are the main terpenoids and are degraded by microorganisms into important flavor and aroma components after harvest and during fermentation (Liang et al., 2012). The content of carotenoid degradation products, such as β-damascenone, megastigmatrienones (4 isomers), α-ionone and β-ionone, is positively correlated with the flavor and aroma of flue-cured tobacco (Leffingwell, 2002). As such, inadequate carotenoid degradation affects aroma formation and the degradation of terpenoids can compensate for the lack of aroma.

Therefore, in view of the quality defects of tobacco leaves, and to promote aroma production in tobacco leaves, this study screened for suitable carotenoid degrading microorganisms from alcoholized tobacco leaves, and adopted suitable fermentation conditions to strengthen the microbial flora on tobacco leaves, accelerate the change of material components in tobacco leaves, and effectively improve tobacco leaf quality.

## 2 Materials and methods

### 2.1 High throughput screening of functional strains

To obtain a greater number of strains from the microbial communities, single cells were isolated by flow cytometry. Different high-throughput screening methods were used to obtain the corresponding functional strains according to the respective screening purposes.

#### 2.1.1 Single cell isolation and culture

A 10 ± 0.1 g sample of non-molting and non-mildew tobacco was selected and added to 200 mL sterilized 0.1 mol/L PBS buffer solution (pH 7.2), and shaken at 220 rpm and 30°C for 2 h. After ultrasonication for 5 min, the mixture was filtered through two layers of sterile degreasing gauze. The filtrate was centrifuged at a low speed (500 × g) for 5 min to separate non-cellular particles, and then centrifuged at a high speed (7,000 × g) for 10 min to collect tobacco microbial precipitates. Next, the precipitate was resuspended in sterile distilled water and diluted to an OD_600_ of 0.3. After staining with 7-AAD dead and living cell dye (BD Pharmingen, New Jersey, United Stated) for 20 min, the obtained suspension was repeatedly washed with sterile distilled water and centrifuged to remove any residual dye. 1 mL of suspension was filtered through a 40 μm filter and injected into the sample box of a flow cytometer (FACSAria III Cell Sorter, BD Biosciences, United Stated). Single cells were allowed to pass through the nozzle and be sorted into 96-well plates. The separation parameters were as follows: Dulbecco‘s phosphate buffer solution, used to protect cells and clean nozzles. Nozzle size was 100 μm; sheath pressure: 30 psi (1 psi = 6.895 kPa), The oscillation frequency was 4,000 Hz and amplitude 30–50 V Sort mode was set to “Single-0.5 Droplet”. The pressure difference between the sample and the sheath was 0.2–0.3 psi to ensure 3,000 to 5,000 cells passed through the nozzle per second. In 96-well plates, 100 μL of different media were injected into each well. The bacteria were cultured in Luria-Bertani medium (LB) at 37°C for 24–72 h. The fungi were cultured in Bengal red medium at 30°C for 72–120 h (Wu et al., 2021).

#### 2.1.2 Selection and acquisition of functional strains

Colonies formed in the 96-well plates were transferred to 96-well plates containing selective media for primary screening by a QPix420 automatic strain selection system. The selective medium for screening α-amylase producing strains contained 10 g/L soluble starch as the sole carbon source in the original proliferation medium. Selective medium for protease-producing strains contained soluble casein 2 g/L instead of peptone. The selective medium for carotenoid-degrading strains used 20% (v/v) tobacco leaf extract (Soak 5 g of tobacco leaves in 100 mL of water, turn 1,200 rpm on a shaker and shake for 2 h) added to the proliferation medium. Those strains that could lighten the color of the tobacco leaf extract were retained. The strains that could grow well on the above selective media or lighten the color of the medium were transferred to 96 deep-well plates and cultured again. Then the enzyme activity of the strains after primary screening was rescreened. Finally, microorganisms with high lipoxygenase (LOX) activity were selected as the research object to investigate the ability of the strains to produce enzymes and improve the quality of tobacco leaves (Robert and Andrew, 2000).

#### 2.1.3 Detection of strain enzyme activity

The activity of α-amylase produced by the strains was studied by DNS (3,5-dinitrosalicylic acid) colorimetric method. One unit (U) of α-amylase activity was defined as the amount of enzyme required to produce 1 mg of reducing sugar per minute at 60°C and pH 6.0. Determination of protein degradation ability of strains was performed according to the Folin phenol colorimetric method to detect protease activity. One activity unit (U) of neutral protease was defined as the amount of enzyme producing 1 μg tyrosine per minute at 40°C and pH 7.0. The determination of LOX enzyme activity, 1 unit of lipoxygenase activity U) was defined as a unit of time, at the wavelength of 234 nm, per milliliter of crude enzyme solution can make the substrate absorbance change of 0.1 for a unit. The substrate was 29 mL PBS solution (pH 6.5) to which was added with 10 μL linoleic acid (final concentration 0.20 mol/L), 5 μL Tween-20 and 1 mL 0.10 mol/L NaOH solution (Xu et al., 2011; Zhang et al., 2014; Li et al., 2021).

#### 2.2.4 Strain identification

Strains were identified by 16 S rDNA or ITS1 gene sequence Bacterial and fungal DNA were, respectively, extracted using MiniBEST bacterial genomic DNA extraction kit and fungal genomic DNA extraction kit according to the manufacturers’ protocols. Polymerase chain reaction (PCR) of the bacterial 16 S DNA sequence was carried out in a 50 μL reaction volume. Amplification was performed using forward primers 27 F (5′-GAG​AGA​GTT​TGA​TCC-TGG​CTC​AG-3′) and 1492 R (5′-ACGGGCGGTGTGTRC-3′). Fungal polymerase chain reaction (PCR) of the ITS1 gene was carried out using the forward primers ITS1 (5′-TCC​GTA​GGT​GAA​CCT​GCG​G-3′) and ITS4 (5′-TCC​TCC​GCT​TAT​TGA​TAT​GC-3′) were used to amplify ITS1-5.8 S rRNA-ITS2 in 50 μL reaction volume. The amplification temperature program for both genes was 5 min at 95°C, 30 cycles of 94°C, 90 s, 55°C, 30 s, and a final extension at 72°C for 2 min. The PCR products were sent to Biotechnology (Shanghai) Co., Ltd (Shanghai, China) for sequencing. The sequencing data were uploaded to the NCBI database (National Center for Biotechnology Information, https://blast.ncbi.nlm.nih.gov/), and compared with identified species using BLAST (Local Alignment Search Tool) to identify the strains (Zhang et al., 2018).

#### 2.2.5 Application effect of functional bacteria to fermented tobacco

Bacteria were seed cultured in Luria-Bertani medium (peptone 10 g/L, yeast powder 5 g/L, sodium chloride 10 g/L) and fungi were seed cultured in Bengal red medium (peptone 5 g/L, glucose 10 g/L, potassium dihydrogen phosphate 1 g/L, magnesium sulfate (MgSO4·7H2O) 0.5 g/L, agar 20 g/L, 1/3000 Bengal red solution 100 mL, chloramphenicol 0.1 g/L). When the concentration of the strain reached 1 × 10^7−8^ CFU/mL, the seed culture requirements were met. The seed culture of the strain was sprayed evenly on the surface of tobacco leaves (fresh flavor style flue-cured tobacco leaf: Sichuan Liangshan Huidong Flue-cured Tobacco C1F in 2018, robust flavor style flue-cured tobacco leaf: Yunnan CXBK in 2019, intermediate aroma style flue-cured tobacco leaf: Guangdong Nanxiong C4F in 2018) with 20% (v/w) inoculation amount. The inoculated tobacco leaves were placed in a constant temperature and humidity incubator for fermentation. Culture conditions were as follows: bacteria 37°C, fungi 30°C, relative humidity 80%, stirring once every 4 h to ensure uniform fermentation. After 1, 1.5, 2, 2.5 and 3 days of culture, we take 50 g samples for each group. Tobacco sampleswere dried at 85°C, and the sensory evaluation was carried out after cutting and rolling to investigate the effect of the strains on the quality of tobacco leaves. The control group was treated with sterile distilled water according to the same conditions and steps outlined above (Peng et al., 2021).

#### 2.2.6 Determination of volatile components

Each sample was ground for 90 seconds at 60 Hz using a grinding machine (TL-48R, Jingxin, Shanghai, China) and sieved through a 6-mesh sieve. 2.00 ± 0.01 g sample powder was thoroughly mixed with 1 μL internal standard (1 μg/mL tritiated naphthalene solution, chromatographically pure, Aladdin) and placed in a 20 mL headspace bottle. Extraction was performed with SPME fiber (DVB/CAR/PDCS, divinylbenzene/carboxyl/polydimethylsiloxane, 50/30 μm) (Supelco, Inc., Bellefonte, PA, United State) at 60°C for 30 min. An Agilent 7890-Pegasus HT GC/MS system and Agilent DB-5MS chromatographic column (30 m × 250 μm × 0.25 μm) (Agilent, United State; lECO, United State) were used to detect volatile components in the samples. The chromatographic conditions were as follows: the flow rate of chromatographic column was 1 mL/min; the inlet temperature was 250°C; 40°C for 2°min, increase 10°C/min to 250°C, 6 min. The ion source adopted the electron bombardment model, and the electron energy was 70 eV. The temperatures of transmission line and ion source were 280°C and 210°C respectively. The mass spectrometry data were obtained at a rate of 3 specs/s at a speed of 33–400 atomic mass units in a full scan mode after a solvent delay of 3°min, and the data acquisition rate was 10 specs/s. With the support of Chroma TOF 4.3X software and LECO-Fiehn Rtx5 database (LECO Corporation), original peak extraction, baseline filtering and calibration, peak alignment, deconvolution analysis, peak recognition and integration were performed. The compounds obtained by peak analysis were matched with the mass spectrometry information in the National Institute of Standards and Technology (NIST, version 1.6) and Wiley library (version 9.0). Identified compounds with a matching score of more than 700 were retained for further analysis. The differential metabolites were screened by removing the relative standard deviation (RSD) of the peak area in the sample >30.0% or detecting 1 and *p*-value < 0.05 (Xia et al., 2022).

#### 2.2.7 Sensory evaluation

Seven smoking personnel with professional smoking qualification were organized to form an evaluation group, and the sensory evaluation of fermented tobacco leaves was carried out according to the standard ’ YC/T138-1998 tobacco and tobacco products sensory evaluation method ' and “YC/T496-2014 cigarette sensory comfort evaluation method” (Shen et al., 2011).

#### 2.2.8 Data processing

All samples were parallel to 3 samples, and the data were expressed as mean ± mean standard error (SEM). The difference of volatile components in samples was analyzed by PCA (principal component analysis) and OPLS-DA (orthogonal partial least squares discriminant). GraphPad Prism 6 (GraphPad Software, San Diego, CA, United State) was used for analysis of variance. Paired *t*-test was used and *p* < 0.05 was considered statistically significant (Ouyang et al., 2022).

## 3 Results

### 3.1 Screening of functional strains

**FIGURE 1:**
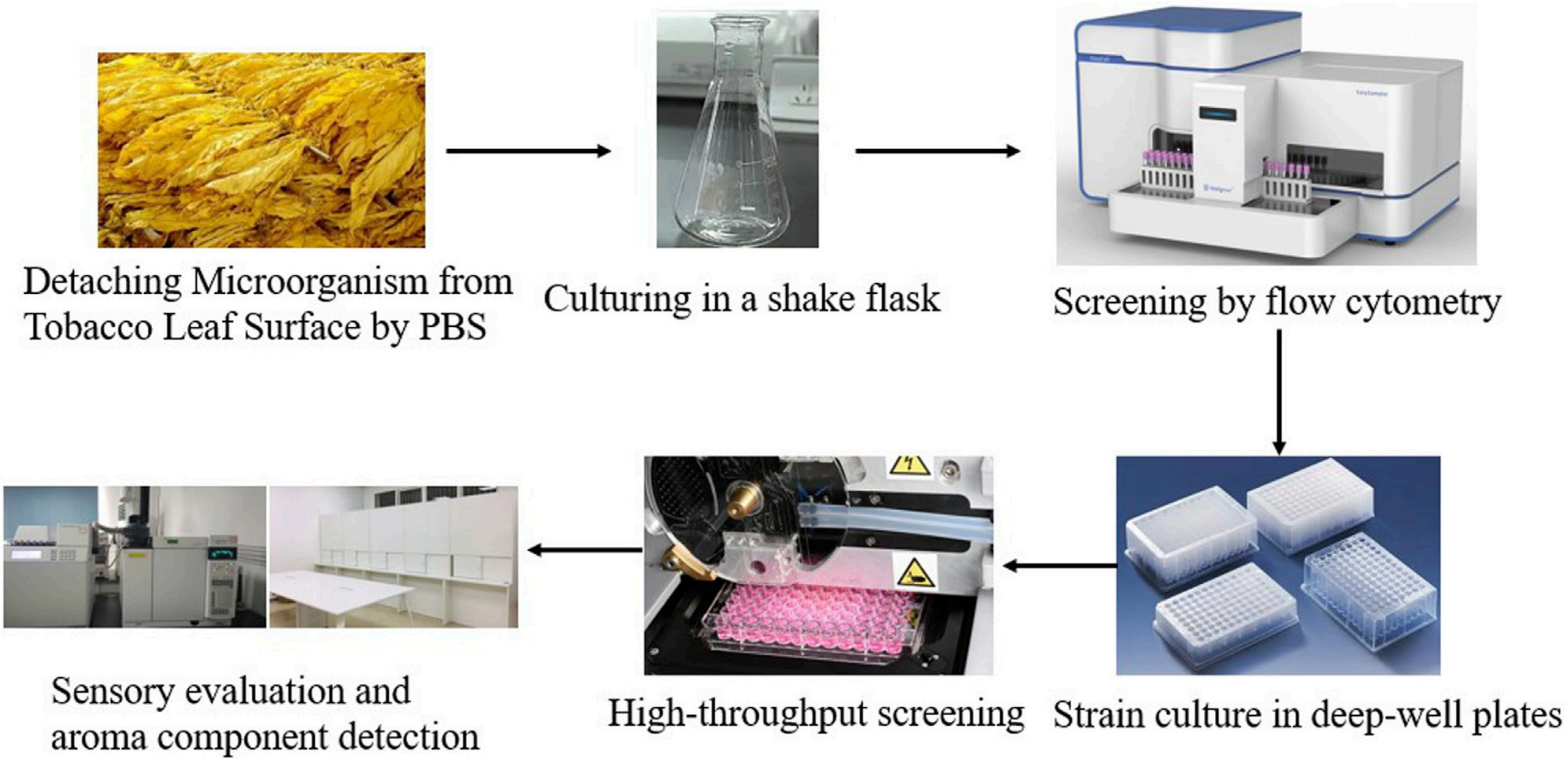
Diagram for high-throughput screening of functional strains.

Through previous research on the prediction and analysis of the function of microbial flora and the analysis of the key compounds that have an important impact on the quality of alcoholized tobacco leaves, it is known that the degradation of the less desirable components of macromolecular substances, such as starch and protein, that affect the flavor is helpful in improving the smoking and eating taste of tobacco leaves, reduce the irritation and miscellaneous gas, promote the formation of flavor substances, and help to highlight the moist, sweet and aroma style of light aroma tobacco leaves. Strains with high LOX enzyme activity contribute to the degradation of carotenoids and the degradation of long-chain fatty acids to promote the formation of aroma substances. Strains for this study were chosen according to their corresponding enzyme production capacity, the ability to improve the quality of tobacco leaves and the growth performance of the strain itself. The technical route of the screening process is outlined in Figure 1.

#### 3.1.1 Screening of functional strains that degrade starch and increase sweetness

**FIGURE 2:**
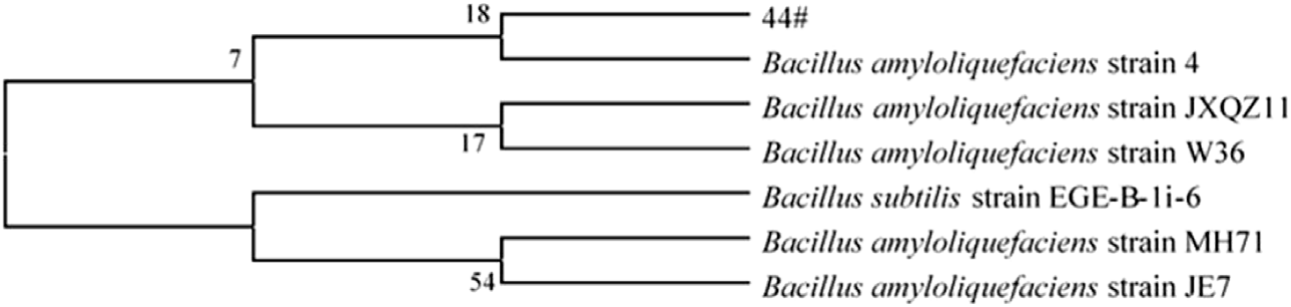
Phylogenetic tree of *B. amyloliquefaciens* 44#.

Through a combination of flow cytometry sorting and high-throughput screening technologies, strains with strong starch degradation ability were obtained from out of more than 1,000 tested strains. It was identified as *Bacillus amyloliquefaciens*. The phylogenetic tree of the strains is shown in Figure 2. Strain 44# (strain preservation number GDMCC No.61457, the sequencing data have been submitted to NCBI, accession numbers: OQ071599) was used for bioaugmented fermentation of tobacco leaves, and the effect of solid-state fermentation was verified. This strain could improve the quality of different flavor tobacco leaves with alcoholization time of up to 2 years, but the quality still did not meet the industry requirements. The sensory evaluation results of tobacco leaves are shown in Table 1, and the changes of total volatile components are shown in Table 2. Strain 44# was helpful for starch degradation and production of reducing sugars, which can increase the sweetness and rich aroma of tobacco leaves, and the strain had a good effect on different aroma types of tobacco leaves. The content of heterocyclic compounds in the composition was greatly affected, which may be due to the strain promoting the production of reducing sugars, thereby contributing to the production of Maillard reaction products.

**TABLE 1:** Sensory evaluation results of 44# strain on low-grade tobacco leaves.

Sample	Sensory evaluation
fresh flavor style flue-cured tobacco leaf	Before fermentation: the overall characteristics of tobacco smoking are obvious irritation and mixed gas. The aroma was faint, but the sweetness was still there, and on the whole has a certain sweetness
	After fermentation: smoke becomes soft and delicate, fragrance becomes more abundant, a little more sweet fragrance, increased sweetness
robust flavor style flue-cured tobacco leaf	Before fermentation: the aroma is faint, there is obvious soil gas wood gas, sweetness is weak. The smoke has a certain degree of clarity, clarity is still there but the strength is not very significant, throat stimulation is obvious
	After fermentation: increased aroma concentration, the strength decreased slightly, the permeability increased, and the rhyme and texture were retained well
intermediate aroma style flue-cured tobacco leaf	Before fermentation: full aroma, texture is good, but the smoke clearness is too large, strength is obvious, the stimulation is too large, taste comfort is poor, the aroma of alkaline atmosphere is slightly obvious, tongue stagnation is more obvious
	After fermentation: strength, irritation, taste comfort are better, aroma quality is retained better effect

**TABLE 2:** Changes of volatile components in tobacco leaves treated by strain 44# (ug/g).

Sample		Alcohols and phenols		Acid	Ester	Carbonyl class	Heterocyclic	Hydrocarbon	*p*-value
fresh flavor style flue-cured tobacco leaf	Before fermentation	213.42 ± 1.06	11.45 ± 0.03		22.01 ± 0.11	167.37 ± 1.12	1099.58 ± 5.66	896.94 ± 2.67	6.36e-06
	After fermentation	248.89 ± 2.13	7.68 ± 0.54		25.93 ± 0.25	227.18 ± 1.99	1487.23 ± 4.63	790.80 ± 3.35	
robust flavor style flue-cured tobacco leaf	Before fermentation	158.89 ± 1.14	14.14 ± 0.21		38.90 ± 0.33	369.58 ± 3.23	609.81 ± 3.32	764.09 ± 4.33	2.79e-06
	After fermentation	163.21 ± 2.14	17.60 ± 0.11		41.15 ± 0.32	379.26 ± 2.45	656.41 ± 2.44	778.13 ± 3.81	
intermediate aroma style flue-cured tobacco leaf	Before fermentation	213.50 ± 1.12	10.65 ± 0.78		20.47 ± 0.29	155.66 ± 1.44	1022.61 ± 3.49	718.82 ± 2.68	3.08e-07
	After fermentation	220.20 ± 2.01	11.39 ± 0.10		18.99 ± 0.01	189.74 ± 1.21	1279.85 ± 4.35	729.89 ± 3.01	

#### 3.1.2 Screening of strains that degrade proteins and reduce irritation

**FIGURE 3:**
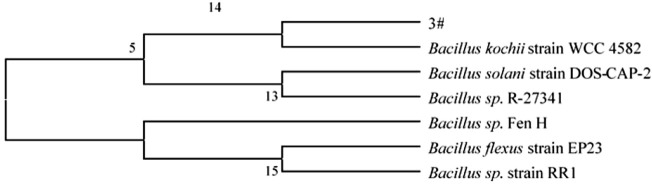
Phylogenetic tree of *B. kochii* 3#.

Through a combination of flow cytometry sorting high-throughput screening technologies, strains with strong protein degradation ability were obtained from more than 1,000 strains. They were identified as *B. kochii*. The phylogenetic tree of the strains is shown in Figure 3. Strain 3# (strain preservation number GDMCC No.61029, the sequencing data have been submitted to NCBI, accession numbers: PRJNA762207) was used for bio-enhanced fermentation of tobacco leaves, and the effect of solid-state fermentation was verified. The strain could improve the quality of different flavor tobacco leaves with alcoholization time of up to 2 years, however the quality could not meet the industry requirements. The sensory evaluation results of tobacco leaves are shown in Table 3. The results of sensory evaluation showed that the strain could increase the aroma concentration of tobacco leaves and help to reduce irritation, but the treatment effect on different aroma types of tobacco leaves varied. The treatment effect on fresh flavor style flue-cured tobacco leaf was the best, and the treatment effect of robust flavor style flue-cured tobacco leaf was worse than that of fresh flavor style and intermediate aroma style flue-cured tobacco leaf. Changes in volatile components of tobacco leaves treated with strain 3# are shown in Table 4.

**TABLE 3:** Sensory evaluation results of strain 3# on low-grade tobacco leaves.

Sample	Sensory evaluation
fresh flavor style flue-cured tobacco leaf	Before fermentation: the whole smoking of tobacco leaves is characterized by obvious irritation and mixed gas, and the aroma was faint. But there was still sweetness. The whole had a certain sweetness
	After fermentation: slightly stimulated, increased aroma concentration, fullness was improved, miscellaneous gas slightly reduced
robust flavor style flue-cured tobacco leaf	Before fermentation: the aroma was dim, there was obvious earthy wood gas, sweetness was weak. The smoke had a certain degree of clarity, but the strength was not very significant, throat irritation
	After fermentation: aroma sweetness was increased, the concentration had increased and enhanced the fullness of the aroma
intermediate aroma style flue-cured tobacco leaf	Before fermentation: full amount of aroma, texture is good, but the smoke was green mixed and gas heavy, strength was obvious. The stimulation was too great, taste comfort was poor, and the aroma of alkaline atmosphere was slightly obvious. Tongue residue was more obvious
	After fermentation: a sense of stimulation, greater momentum, limited improvement

**TABLE 4:** Changes of volatile components in tobacco leaves treated by strain 3# (ug/g).

Sample		Alcohols and phenols	Acid	Ester	Carbonyl class	Heterocyclic	Hydrocarbon	*p*-value
fresh flavor style flue-cured tobacco leaf	Before fermentation	155.36 ± 1.24	16.35 ± 0.23	38.66 ± 1.01	158.59 ± 1.26	887.78 ± 3.44	783.26 ± 1.02	8.50e-06
	After fermentation	189.54 ± 2.12	10.26 ± 0.31	28.89 ± 0.34	179.89 ± 2.21	799.65 ± 2.49	644.52 ± 3.20	
robust flavor style flue-cured tobacco leaf	Before fermentation	135.63 ± 2.44	15.69 ± 0.22	55.96 ± 0.41	336.56 ± 2.94	652.31 ± 1.55	874.14 ± 1.00	3.71e-08
	After fermentation	167.58 ± 3.01	9.60 ± 0.30	50.25 ± 1.01	388.54 ± 3.67	637.89 ± 3.02	858.29 ± 1.21	
intermediate aroma style flue-cured tobacco leaf	Before fermentation	223.37 ± 1.11	20.36 ± 1.43	18.65 ± 0.44	149.67 ± 0.12	997.85 ± 1.22	786.55 ± 2.01	3.08e-07
	After fermentation	310.02 ± 2.92	17.69 ± 0.01	17.33 ± 0.23	132.25 ± 1.02	1026.34 ± 3.20	723.31 ± 3.21	

#### 3.1.3 Screening of strains that promote the production of aromatic substances

**FIGURE 4:**
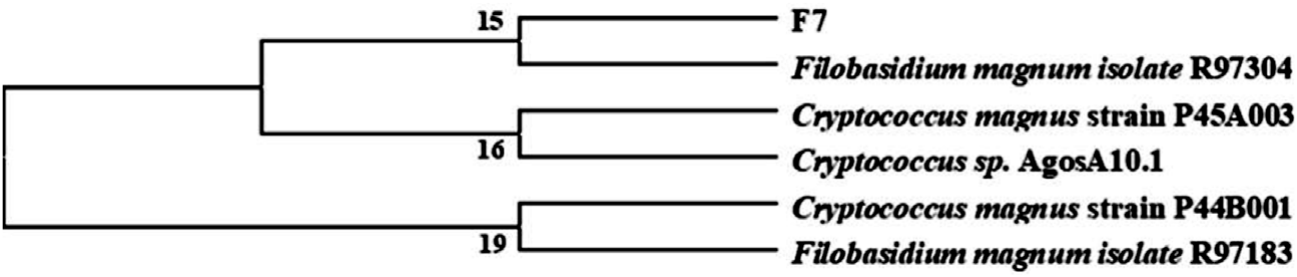
Phylogenetic tree of *F. magnum* F7#.

Through a combination of flow cytometry sorting and high-throughput screening technologies, more than 1,000 strains were examined to obtain a single strain that could cause lightening of tobacco leaf extract color, and through re-screening, it was learned that this strain also possessed a high lipoxygenase enzyme production ability. It was identified as *Filobasidium magnum*. The phylogenetic tree of the strain is shown in Figure 4. The strain was named F7# (strain preservation number GDMCC No.62307, the sequencing data have been submitted to NCBI, accession numbers: PRJNA764574) and was used for bio-enhanced fermentation of tobacco leaves, and the effect of solid-state fermentation was verified. The results of tobacco leaf sensory evaluation are shown in Table 5. The results showed that the strain had obvious effect on aroma enhancement, with special aroma and comfortable aftertaste. Changes in volatile components of tobacco leaves treated with strain F7# are shown in Table 6.

**TABLE 5:** Sensory evaluation results of strain F7# on low-grade tobacco leaves.

Sample	Sensory evaluation
fresh flavor style flue-cured tobacco leaf	Before fermentation: Miscellaneous obvious, stimulating feeling obvious, sweet feeling, aroma dim
	After fermentation: There was a special fragrance, brighter, more comfortable, other indicators to improve also good
robust flavor style flue-cured tobacco leaf	Before fermentation: Aroma dull, heavy wood gas, sweetness was weak, a certain stimulation throat
	After fermentation: Aroma increased, sweetness increased, delayed concentration has a certain increase in delayed fullness
intermediate aroma style flue-cured tobacco leaf	Before fermentation: full aroma, good texture, green miscellaneous gas weight, strength stimulation was greater, slightly uncomfortable aftertaste, oral residue
	After fermentation: reduced stimulation, sweetness increased slightly, the remaining changes were not obvious

**TABLE 6:** Changes of volatile components in tobacco leaves treated by strain F7# (ug/g).

Sample		Alcohols and phenols	Acid	Ester	Carbonyl class	Heterocyclic	Hydrocarbon	*P*-value
fresh flavor style flue-cured tobacco leaf	Before fermentation	193.36 ± 1.23	10.32 ± 0.11	31.26 ± 0.21	167.37 ± 2.01	896.35 ± 4.03	754.36 ± 2.39	1.10e-05
	After fermentation	215.57 ± 2.22	5.32 ± 0.02	25.54 ± 0.11	227.18 ± 2.24	766.98 ± 3.99	658.84 ± 4.02	
robust flavor style flue-cured tobacco leaf	Before fermentation	132.21 ± 0.34	13.39 ± 0.01	53.69 ± 1.24	325.37 ± 3.21	616.35 ± 2.34	896.34 ± 3.98	1.36e-07
	After fermentation	153.29 ± 1.51	11.26 ± 0.01	51.33 ± 1.34	384.26 ± 2.98	652.45 ± 3.02	858.29 ± 3.20	
intermediate aroma style flue-cured tobacco leaf	Before fermentation	208.98 ± 1.25	21.88 ± 0.10	17.79 ± 0.45	158.00 ± 1.32	992.01 ± 4.38	777.39 ± 2.33	4.98e-06
	After fermentation	312.25 ± 2.57	16.65 ± 0.47	16.26 ± 1.01	141.26 ± 1.01	1132.00 ± 3.39	716.25 ± 3.24	

### 3.2 Optimization of solid-state fermentation time

**FIGURE 5:**
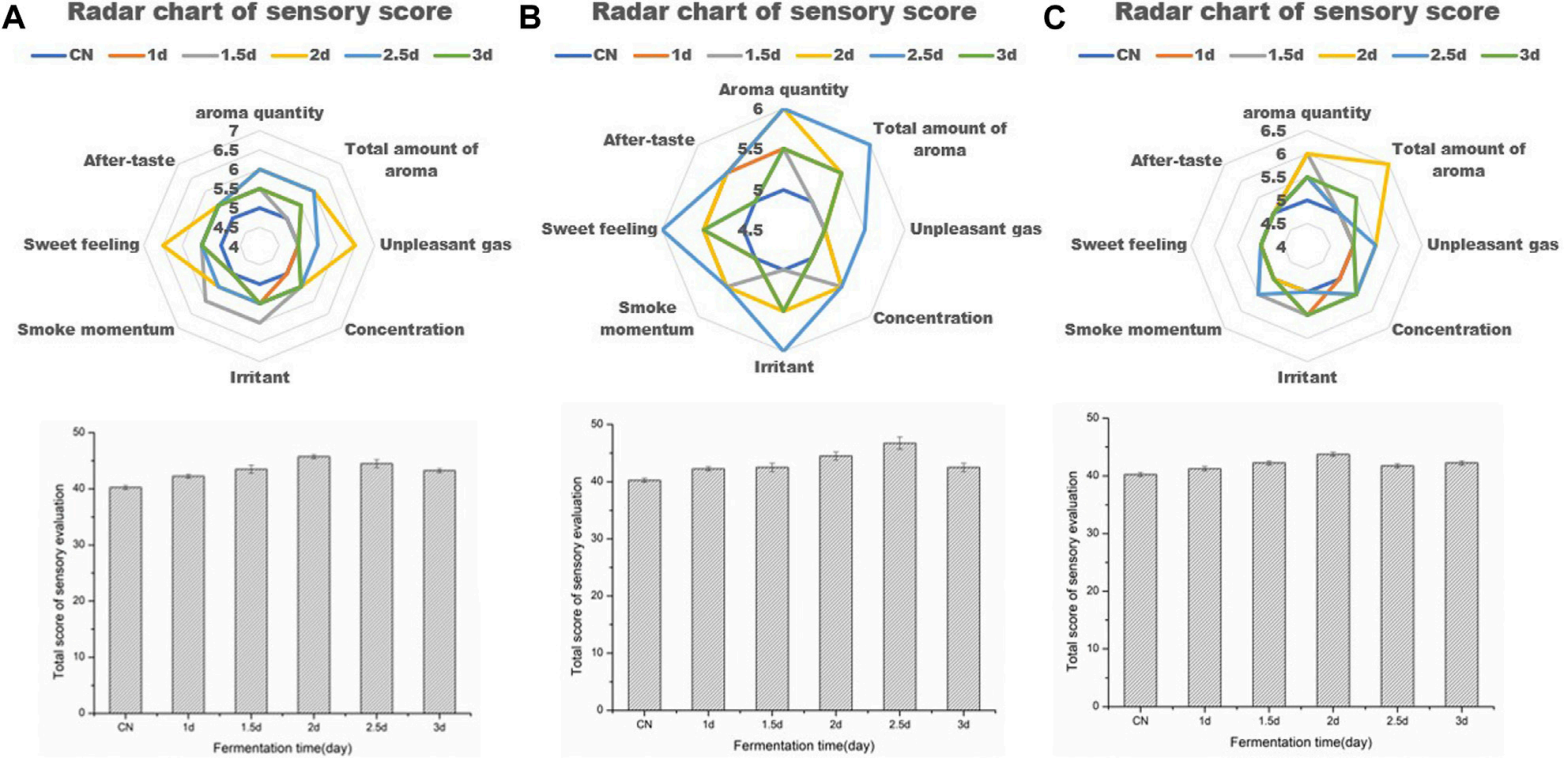
Sensory evaluation results of tobacco leaves fermented with functional strains for different time periods **(A)**. Optimization of fermentation days with strain 44# **(B)**. Optimization of fermentation days with strain 3# **(C)**. Optimization of fermentation days with strain F7#.

In order to better verify the quality improvement effect of functional strains on tobacco leaves, the aspect of solid-state fermentation time on the fermentation effect was studied. Sensory evaluation was carried out on samples from 0, 1, 1.5, 2, 2.5 and 3 d after fermentation. The results showed that strain 44# at 2 d, strain 3# at 2.5 d, and strain F7# at 2 d could obtain better treatment effects (Figure 5). However, the quality improvement effect of each strain was different. Strain 44# had a good effect on increasing sweetness, aroma and quality. Compared with the control (CN), the sensory score increased by 7 points reaching 47 points. While strain 3# had a good effect on reducing irritation and increasing softness, and in comparison with CN, the sensory score increased by 6 points reaching 46 points. Strain F7# had a prominent effect on increasing aroma volume and aroma quality, and the sensory score increased by 3.5 points compared with CN reaching 43.5 points. Therefore, in order to improve the quality of tobacco leaves with different defects, the strains could be combined according to their characteristics to achieve a comprehensive quality improvement effect on the tobacco leaves.

### 3.3 Functional strains compounding

**FIGURE 6:**
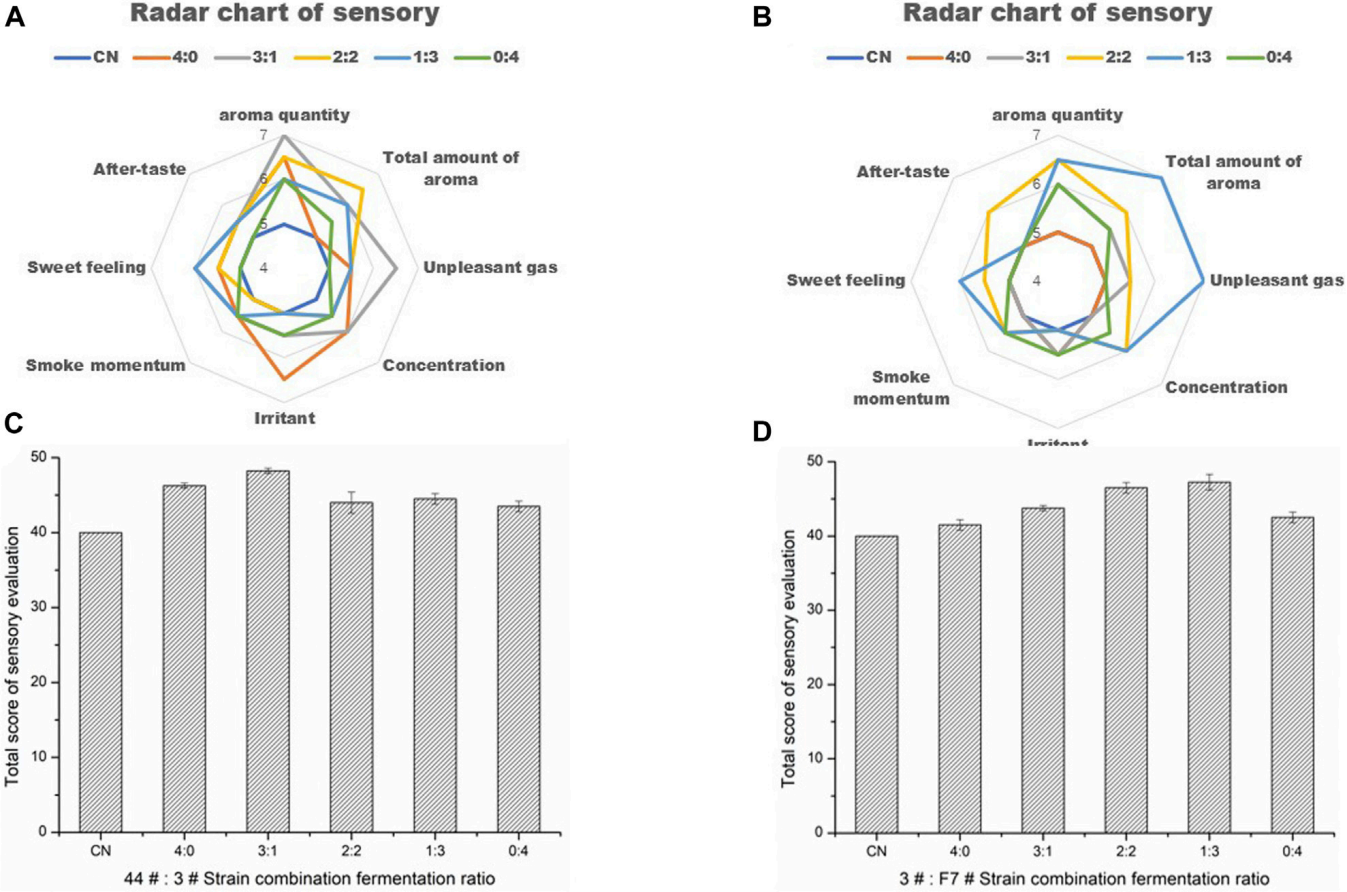
Optimization and sensory evaluation results of combined strains **(A)**. The quality improvement effect of strains 44# and 3# combined was different from that of individual strains **(B)**. The combined effect of strains F7# and 3# was different from that of the individual strains **(C)**. Optimization of the ratio of strains 44# and 3# **(D)**. Optimization of the ratio of strains F7# and 3#.

According to the functional characteristics of the screened strains, they were combined and inoculated to Yunnan CXBK tobacco leaves for fermentation under the condition of a constant total inoculation amount. From the perspective of increasing the sweetness and aroma of tobacco leaves, reducing offensive odor and irritation, and promoting Maillard reaction products, strains 44# and 3# were combined, while in order to reduce the irritation and increase the fragrance, strains F7# and 3# were combined, as shown in Table 7. The optimization of strain combination ratio is shown in Figure 6. The optimal combination ratio of strains 44# and 3# was 3:1, and the optimal ratio of strains F7# and 3# was 1:3. After fermentation for 48 h, the sensory evaluation results showed that the individual strains and the combined cultures were better than CN, increasing the sensory score by seven to eight points. The inoculation of tobacco leaves with strains 3# and F7# promoted the aroma-enhancing effect of F7# strain, producing a unique aroma, improved aroma quality, and increased sweet aroma.

**TABLE 7:** Function and combination of each strain.

Strain	Generic name	Function	Sensory evaluation	Preferred combination scheme
44#	*B. amyloliquefaciens*	High amylase yield, degraded starch	The 44 # strain bio-enhanced fermentation of tobacco leaves for 2 days, compared with the control tobacco sensory score increased by 7 points; effect description: ‘Aroma improvement, tobacco quality improvement'	1) The total inoculation amount of 44#:3# was 20%, and the sensory score was increased by 8 points compared with the control tobacco leaves after fermentation for 2 days at the ratio of 3 : 1
3#	*B.acillus kochii*	High protease production, protein degradation, contribute to the formation of aromatic compounds	Strain 3# of tobacco leaves for bio-enhanced fermentation 2.5 days, compared with the control tobacco sensory score increased by 6 points; effect description: ‘Tobacco sweetness increased, aroma increased significantly'	Effect description: ‘further enhance the aroma, irritation reduced’. 2) The total inoculation amount of F7#:3# was 20%. After fermentation for 2 days, the sensory score of tobacco leaves was increased by 7 points compared with that of control tobacco leaves. Effect description were ‘aroma texture is better, sweet, obvious, aroma texture is better'
F7#	*Filobasidium magnum*	Lipoxygenase is relatively high, which contributes to the formation of carotenoids and long-chain fat degradation products	Strain F7# was used for bioaugmented fermentation of tobacco leaves for 2 days, and the sensory score was increased by 3.5 points compared with the control tobacco leaves. Effect description: “Aroma is rich, aftertaste is comfortable, irritation is reduced.”	

### 3.4 Volatile component analysis

**FIGURE 7:**
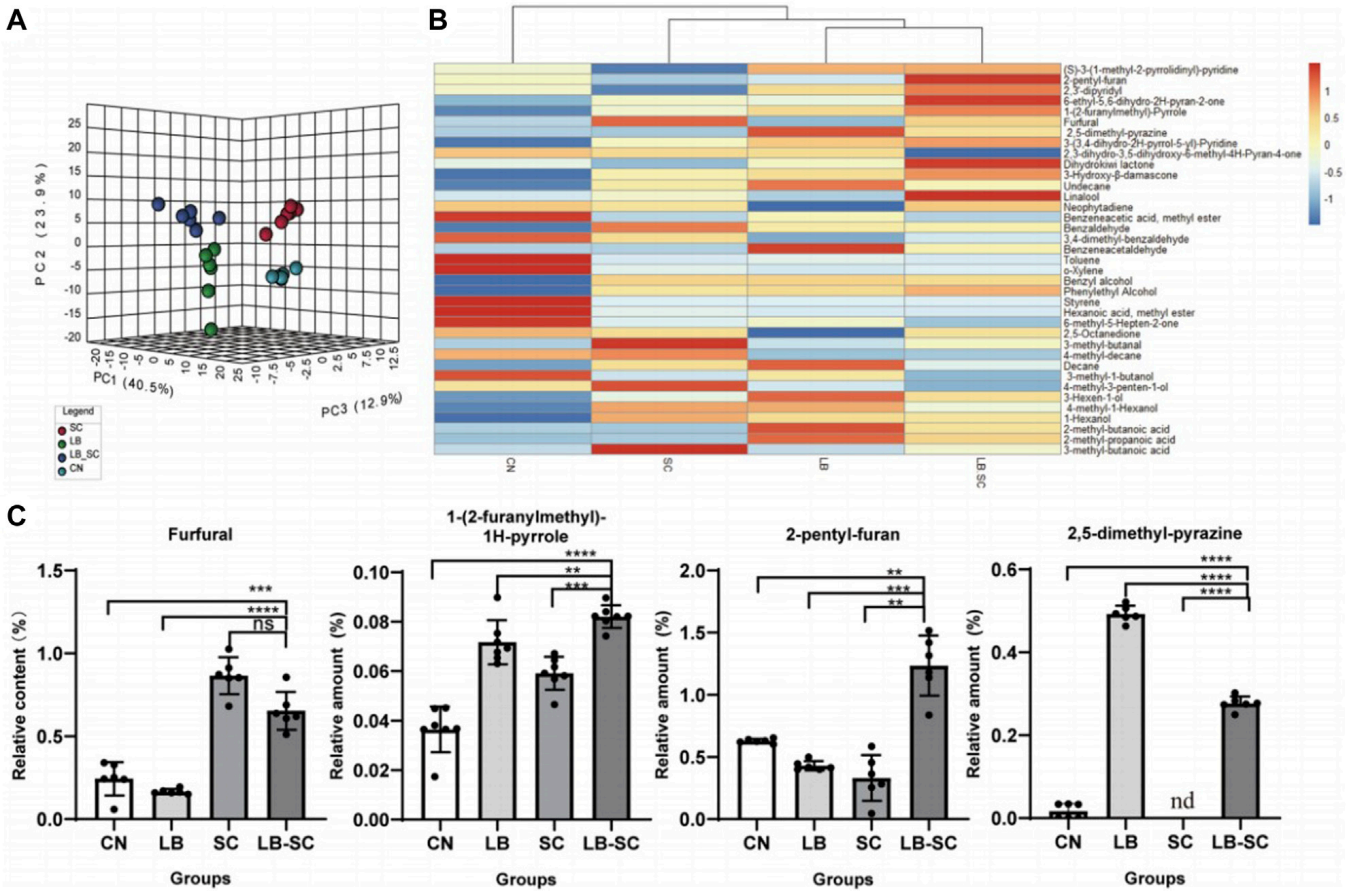
Optimization of volatile component analysis by combination of strains four# and 3# **(A)** PCA analysis of compound components from combined culture of strains 44# and 3#.**(B)**. Heat map analysis of compound and differential components of individual strains **(C)**. For the analysis of the content of some odorant components, LB was used for pure culture of strain 44#, SC for pure culture of strain 3#, and LB-SC for 44#-3# combined culture.

Non-targeted metabolomics technology based on HP-SPME-GC/MS was used to analyze the volatile components from the biological fermentation samples of strains 44# and 3#, both individually as pure cultures and as a combined culture. A total of 163 compounds were detected, including 40 heterocyclic compounds, 38 carbonyl compounds, 28 alkanes or olefins, 22 alcohols or phenols, 12 esters, 13 benzenes and 6 acids and others. The PCA three-dimensional scatter plot based on the relative content of volatile components showed that the first three axes respectively explained 40.5%, 23.9% and 12.9% of the total variance (Figure 7A), indicating a significant difference in sample composition. The spread of data distribution showed that there were significant differences in the composition of tobacco leaves between the biological fermentation group and the control group. In addition, in the scatter plot of the OPLS-DA model, all samples were within the 95% confidence interval. Through 200 response permutation tests, the OPLS-DA model was effective, did not show overfitting, and had good predictive performance. The 38 differential compounds including 10 heterocyclic compounds, seven carbonyl compounds, 8 alcohols, 3 alkanes, 2 olefins, 3 acids, 2 esters and 3 aromatic compounds screened by VIP and *p* values (Figure 7B). According to the substances produced, 4 compounds were related to the metabolism of terpenoids, 9 were related to phenylalanine degradation, and 10 were related to Maillard reaction. Reducing sugars and amino acids produce a series of Maillard reaction products under certain conditions. Amadoury rearrangement forms furfural and its derivatives, reduction of ketone heterocyclic formation of pyrazine, furan, pyrrole and other heterocyclic compounds (Gong et al., 2021). The biological fermentation of strain 44# combined with that of strain 3# provided a basis for promoting Maillard reaction. The content of Maillard reaction products in the combined culture was higher than that in the individual cultures and control group. The contents of 2-pentylfuran and 1-(2-furylmethyl)-1H-pyrrole were significantly higher, increasing by 185% and 212%, respectively, compared with the control, reaching 1.82 ug/g and 25.86 ug/g. Although the furfural content in combined culture was not the highest, there was no significant difference between the pure cultures and combined culture, and the combined fermentation showed an increase of 325% compared with the control group, reaching 19.57 ug/g. The content of 2,5-dimethylpyrazine was higher in the pure culture fermentation of strain 44# and the combined fermentation samples, while it was very low in the strain 3# pure culture fermentation samples, and the lowest in the control group (Figure 7C). Among terpenoid metabolites, dihydroactinidiolide, linalool, 3-hydroxy-β-damascenone and neophytadiene had the highest content. The content of most alcohols and acids were highest in samples fermented with individual strains. Among the benzene substances, benzyl alcohol, phenylethyl alcohol and phenylacetaldehyde were higher, while other benzene substances were degraded or converted during culture growth. The contents of main flavor compounds of strain combinations 4# and 3# are shown in Table 8.

**TABLE 8:** Main differential flavor compounds content of combination of strains 4# and 3# (ug/g).

Sample	Furfural	1-(2-furylmethyl)-1H-pyrrole	2-Pentylfuran	2,5-Dimethylpyrazine
CN	6.02 ± 0.21	0.86 ± 0.32	13.98 ± 0.01	0.24 ± 0.01
44#	4.74 ± 0.04	1.87 ± 0.29	11.71 ± 0.08	13.38 ± 0.26
3#	15.75 ± 0.34	1.07 ± 0.23	5.98 ± 0.35	nd
44#-3#	19.57 ± 0.40	1.82 ± 0.31	25.86 ± 0.64	6.24 ± 0.13

**FIGURE 8:**
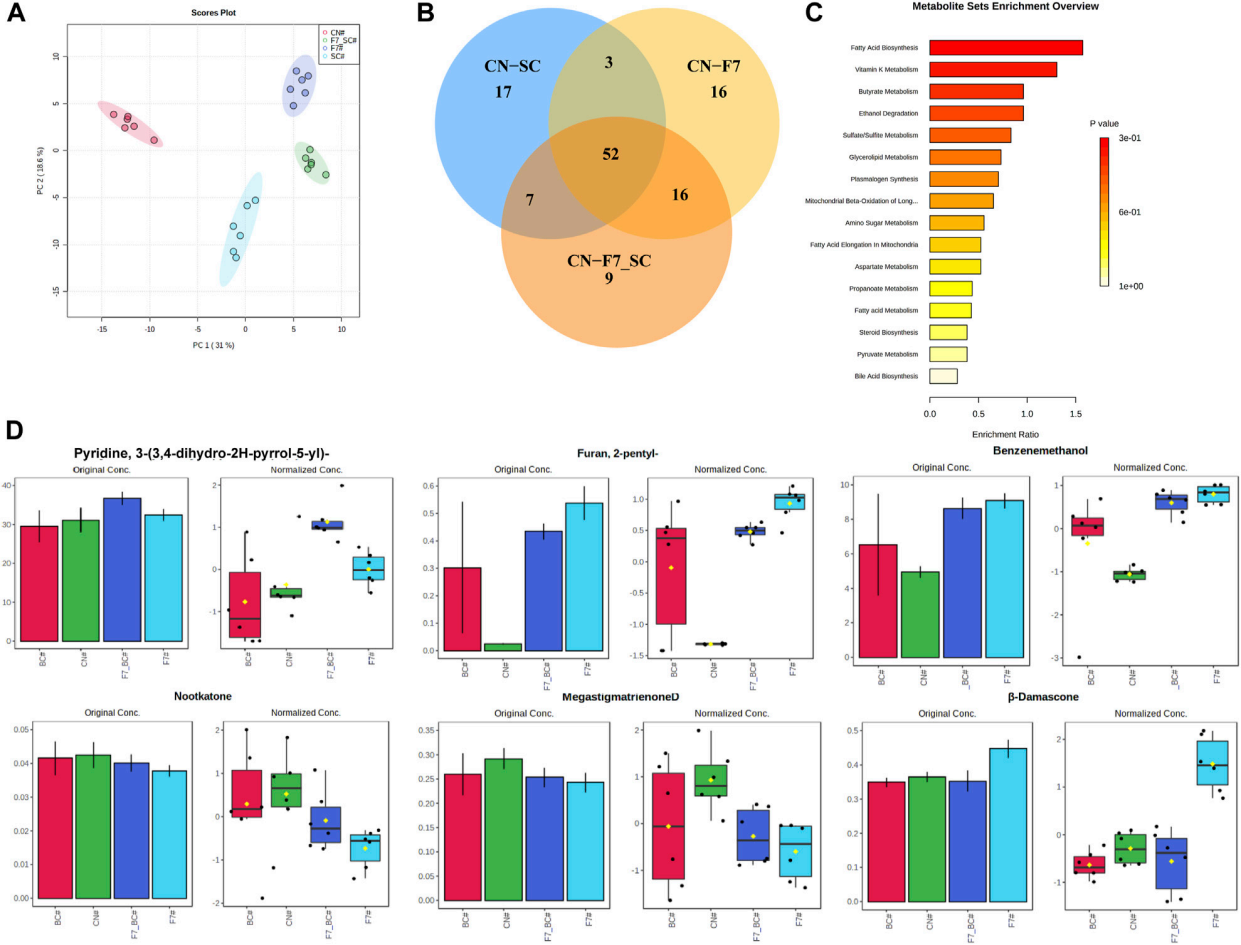
Optimization of compound culture of strains F7# and 3# **(A)** PCA analysis of molecular compounds from strains F7# and 3# **(B)**. Analysis of common differences between the combined culture and the pure cultures **(C)**. Differential metabolic pathways reflected by the differential metabolites **(D)**. Analysis of some aromatic components. SC was strain 3#, and F7-SC was F7#-3# combined culture.

A total of 191 volatile compounds were detected from the pure culture fermentation and combined bio-fermented tobacco samples of strains F7# and 3#, including 35 heterocyclic compounds, 32 ketones, 27 esters, 19 aldehydes, 13 alkanes and olefins, 14 aromatic hydrocarbons, 11 alcohols, 9 acids and 3 phenols. Using unsupervised principal component analysis (PCA) (Figure 8A), samples could be well distinguished within a 95% confidence interval. The high concentration of sample points on the scatter plot indicates the similarity of sample characteristics and the reproducibility of sample treatment. The two principal components accounted for 50.9% of the total variance. The control and biological fermentation groups displayed obvious differences. Three OPLS-DA models were constructed between control group (CN) and pure culture fermentations (3# and F7#), and combined culture fermentation (F7#-3#). After 1,000 permutation tests, the OPLS-DA model did not overfit and had good predictive ability. The R2Y and Q2 calculated by cross validation were close to 1. According to VIP >1.0 and *p* < 0.05, 87 differential metabolites were obtained between CN and strain 3# pure culture fermentation, 84 differential metabolites were obtained between controlCN and strain F7# pure culture fermentation, and 79 differential metabolites were obtained between CN and the F7#-3# combined fermentation (Figure 8B). The common differential metabolites between the three models included 52 compounds, including 15 esters, 8 carbonyl compounds, 10 heterocyclic compounds, etc. There were 12 kinds of long chain fatty acids, alkanes and lipids related to fatty acid metabolism, including linolenic acid methyl ester, pentadecanoic acid methyl ester, tetradecanoic acid methyl ester, palmitic acid methyl ester, hexadecanoic acid (palmitic acid), dodecane and so on. Aromatic esters (such as formic acid, benzyl ester, acetic acid, phenylacetic acid, ethyl ester) are associated with phenylalanine degradation. The heterocyclic compounds were mostly Maillard reaction products, such as methylpyrazine, 2-pentylfuran, tetramethylpyrazine, etc. Twelve compounds were related to the degradation of terpenoids, such as cembratrienone, megastigmatrienone, 4-heptenal, 2,4-heptadienal, etc. Through the analysis of the metabolic pathways reflected by the common differential metabolites (Figure 8C), the number of differential metabolites in fatty acid metabolism was the highest after treatment. Compared with the control group, the samples treated with individual functional strains, and the F7#-3# combined culture showed the strongest ability to increase or decrease differential metabolites. The relative abundance of some differential metabolites is shown in Figure 8D. The main differences in the content of flavor substances between strain combinations F7# and 3# are shown in Table 9. The content of 3-(3,4-dihydro-2H-pyrrole-5-yl) pyridine in the tobacco leaves treated with the combined culture was 183% of the content in the control sample, reaching 123.52 ug/g. The analysis of the substances that have a key effect on the flavor showed that they played an important role in improving the overall quality of tobacco leaves. The content of benzyl alcohol in tobacco leaves after treatment with the combined culture was 27.36 ug/g and 183% of that in the control sample. 2-Pentyl-furan was the product of Maillard reaction, and its content was 2184% of the control sample, reaching 60.72 ug/g. Meanwhile megastigmatrienone D, an isomer of the aroma component megastigmatrienone, was 83% of the control sample, and this decrease may have been due to its conversion to other isomers or further degradation. As a terpenoid, nocardone is an important precursor of tobacco aroma substances, and its content was reduced by 88% compared with the control group. β-damascenone, an important carotenoid degradation product, increased by 22% compared with the control group, reaching 10.28 ug/g.

**TABLE 9:** Main differential flavor compounds content of combination of strains F7# and 3# (ug/g).

Sample	3-(3,4-Dihydro-2H-pyrrole-5-yl) pyridine	2-Pentyl-furan	Benzenemethanol	Nootkatone	Megastigmatrienone D	β-damascenone
CN	67.50 ± 1.23	2.78 ± 0.03	14.95 ± 1.00	1.01 ± 0.01	6.27 ± 0.43	8.43 ± 0.03
3#	87.20 ± 2.12	7.23 ± 0.12	13.38 ± 1.34	1.25 ± 0.21	7.81 ± 0.03	10.03 ± 1.05
F7#	62.6 ± 1.01	10.25 ± 0.33	16.33 ± 1.01	0.72 ± 0.03	5.34 ± 0.31	8.55 ± 0.67
F7#-3#	123.52 ± 1.15	60.72 ± 0.12	27.36 ± 0.35	0.88 ± 0.01	5.52 ± 0.22	10.28 ± 0.95

## 4 Discussion

In our study, flow cytometry sorting combined with high-throughput screening technology of more than 1,000 microbial strains from the surface of tobacco leaves resulted in the isolation of three strains with significant improvement effect on the quality of tobacco leaves. The characteristics of the three strains were explored by sensory evaluation. Strain 44# aided in starch degradation and reducing sugar production, which was beneficial to increasing the sweetness and rich aroma of tobacco leaves, and had positive effects on different aroma types (Ma et al., 2022). The content of heterocyclic compounds in the composition was greatly affected, possibly because the strain can promote the production of reducing sugars, thereby contributing to the production of Maillard reaction products (Wang et al., 2019). Strain 3# was able to significantly reduce the irritation of tobacco leaves due to its effectively degradation of protein in tobacco leaves (Cai et al., 2022). Strain F7# had a higher lipoxygenase enzyme production capacity, resulting in the production of more flavor substances. When combined, strains 44# and 3# increased the sweetness, while making the smoke obvious, and aroma more mellow. This occurred because the combination of strains was more conducive to the production of Maillard reaction products, promoting the aroma of alcohol and feeling. The combination of strains improved the quality of tobacco leaves better than either strain on its own, which resulted in a more uniform and comprehensive improvement of each index. Combination of strains F7# and 3#, increased the aroma and improved the quality of aroma, significantly increased sweet feeling, and had a special charm. However, in the evaluation and analysis of tobacco leaves, because the samples were sent in batches for evaluation, the score of tobacco leaves was affected by the environmental conditions and the subjective judgment of the evaluators to a certain extent, resulting in the score of the F7#:3# combination being lower than that of individual strain fermentations from different batches, but compared with the quality improvement effect of individual strains evaluated in the same batch, the score of quality improvement was the highest. In addition, in order to verify the effect of tobacco improvement, the volatile components in tobacco samples after solid-state fermentation were further analyzed.

The results showed that the main flavor-related compounds changed more significantly after the use of combined culture fermentation than after fermentation with individual strains. Among the products of Maillard reaction, furfural is a characteristic flavor substance, which can lead to the change of flavor, texture and color of food (Jian et al., 2020). Furfural is common in coffee, baked malt, herbs and other beverages. 2,5-Dimethylpyrazine is a compound found in cocoa, herb and medicinal spices. 2-Pentylfuran is the main aroma contributor in tobacco, with fruit, roasted and sweet aromas (Zhang Z. Y. et al., 2022). 1-(2-furylmethyl)-pyrrole is the main Maillard reaction product of cysteine and ribose, with coffee, green, and hay flavor. In tobacco, terpenoids and their degradation products are also very important flavor substances, especially the degradation products of carotenoids (Cao et al., 2022; Lewinsohn et al., 2005). Linalool is a monoterpene with tetrahydrofuran structure. It is a sweet and earthy compound with floral and fruity flavor. Dihydroactinidiolide is one of the important degradation products of carotenoids (Li et al., 2022). It has a deep and mild odor that can cover up the bad taste of tobacco and improve tobacco’s palatability. 3-Hydroxy-β-damascenone is also a major aromatic compound, with strong fruity, woody and violet aromas and is biotransformed by some microorganisms (Liu et al., 2021). In addition, the phenylalanine degradation products phenethyl alcohol is the characteristic odor of fresh roses, and benzaldehyde has the characteristic odor of bitter almond oil. In summary, the effect of improving tobacco quality by strain combination fermentation is stronger than that by fermentation with any individual strain. The improvement of tobacco leaf quality is closely related to the change of the content of various aroma components, and the increase of aroma components with significant changes was far more than expected.

## 5 Conclusion

In this study, we aimed to ameliorate common quality defects of tobacco leaves, in order to reduce the irritation of tobacco leaves and increase the sweetness, by developing a screening process for the detection and isolation of functional strains for degrading polymer substances. In this study three functional strains, capable of degrading high molecular substances (starch and protein) and promoting the production of aroma substances, which could improve the quality of tobacco leaves were obtained by our high-throughput screening method. *B. amyloliquefaciens* 44#, *B. kochii* 3# and *F. magnum* F7#. The genera to which the three strains belong are common on tobacco leaves of different grades and aroma types during the process of alcoholization, which indicates that their influence on tobacco leaf quality may be universal. *B. amyloliquefaciens* 44#, with its high starch degradation ability, could improve the quality of tobacco leaves with different aroma types, and had a good effect on increasing sweetness, aroma quantity and quality. *B. kochii* 3#, with higher protein degradation ability, was able to reduce irritation and increase softness. *F. magnum* F7#, with its higher lipoxygenase had a great effect on increasing aroma quantity and quality. Each functional strain was subjected to solid-state fermentation of tobacco leaves by bioaugmentation, and the best quality improvement effect was obtained after 2–2.5 days of fermentation. According to the functional differences of each strain, a combined fermentation scheme was designed to achieve the purpose of improving the quality and efficiency of tobacco leaves. Based on increasing the sweetness, reducing irritation and promoting the formation of Maillard reaction products, Strains 4# and 3# were combined to obtain the best quality improvement effect when the two strains were fermented together at a ratio of 3:1. Based on the perspective of reducing irritation and increasing aroma quality, strains F7# and 3# were combined. When these two strains were in a 3:1 ratio, the improvement of each quality index was higher than under either strain by itself. The tobacco leaves fermented by the combination of strains 44# and 3# significantly increased the Maillard reaction products 2-pentylfuran, 1-(2-furanmethyl)-1 h-pyrrole, furfural, 2,5-dimethylpyrazine, etc., by more than 2 times. The increase of 3- (3,4-dihydro-2H-pyrrole-5-yl) pyridine, β-damascenone and benzyl alcohol in tobacco leaves fermented by the combination of strains F7# and 3# was more than 1 time. The increase in 2-pentyl-furan was particularly significant, up to 20 times. At present, the research on mixed fermentation is still in the laboratory stage. Subsequent industrial verification can be carried out through further pilot fermentation, and finally the application in industrial production can be realized, which can truly solve the problems faced by enterprises.

## Data Availability

The original contributions presented in the study are included in the article/supplementary material, further inquiries can be directed to the corresponding author.
